# Relationship Between C-Peptide Levels, Clinical Features, and Serum Data in a Brazilian Type 1 Diabetes Population with Large Variations in Genomic Ancestry

**DOI:** 10.3390/ijms252011144

**Published:** 2024-10-17

**Authors:** Rossana Sousa Azulay, Vandilson Rodrigues, Débora Cristina Ferreira Lago, Ana Gregória Ferreira Pereira de Almeida, Joana D’Arc Matos França de Abreu, Lincoln Matos, Caio Andrade, Gilvan Cortês Nascimento, Marcelo Magalhães, Alexandre Facundo, Clariano Pires de Oliveira Neto, Adriana Guimarães Sá, Dayse Aparecida Silva, Marília Brito Gomes, Manuel dos Santos Faria

**Affiliations:** 1 Service of Endocrinology, University Hospital of the Federal University of Maranhão (HUUFMA/EBSERH), São Luís 65020-070, Brazil; debora.lago@ufma.br (D.C.F.L.); agfpalmeida@gmail.com (A.G.F.P.d.A.); joana-franca@hotmail.com (J.D.M.F.d.A.); lincolnmatos@hotmail.com (L.M.); gilvancortes@uol.com.br (G.C.N.); alexandrenfacundo@hotmail.com (A.F.); clarianoneto@gmail.com (C.P.d.O.N.); mfaria1949@gmail.com (M.d.S.F.); 2 Research Group in Endocrinology and Clinical and Molecular Metabolism (ENDOCLIM), Sao Luis 65020-070, Brazil; vandilson.rodrigues@ufma.br (V.R.); dr.caioanascimento@hotmail.com (C.A.); magalhaes_ms@yahoo.com.br (M.M.); adrianabeckman2024@gmail.com (A.G.S.); 3 Post-Graduate Program in Adult Health (PPGSAD), Federal University of Maranhão (UFMA), São Luis 65085-580, Brazil; 4 DNA Diagnostic Laboratory (LDD), Rio de Janeiro State University (UERJ), Rio de Janeiro 20550-900, Brazil; dayse.a.silva@gmail.com; 5 Diabetes Unit, Rio de Janeiro State University (UERJ), Rio de Janeiro 20551-030, Brazil; mariliabgomes@gmail.com

**Keywords:** type 1 diabetes, C-peptide, microvascular complication, genomic ancestry

## Abstract

Type 1 diabetes (T1D) is a chronic disease characterized by the immune-mediated destruction of the pancreatic beta cells responsible for insulin production. The secreted insulin and C-peptide are equimolar. Due to its longer half-life, C-peptide has become a safer means of assessing the pancreatic reserve. C-peptide levels were evaluated in a population of patients with T1D, focusing on the relationship between this variable and other factors. In addition, the influence of C-peptide on metabolic control and microvascular complications was investigated. This cross-sectional study included 95 patients who had been diagnosed with T1D at least five years earlier. These patients were evaluated using a clinical demographic survey, anthropometric data, laboratory tests, and fundoscopy. This study showed that 29.5% of patients had residual insulin secretion, which correlated directly with their age at diagnosis. No statistically significant differences in metabolic control or microvascular complications were observed between the C-peptide level groups. In addition, our results indicate that ancestry does not influence the persistence of residual C-peptide function in our highly mixed population. It is recommended that future research consider incorporating new variables, such as HLA and pancreatic autoimmunity, as factors that may influence residual β-cell function.

## 1. Introduction

Type 1 diabetes mellitus (T1D) is a chronic disease characterized by the immune-mediated destruction of pancreatic beta cells, resulting from a combination of genetic predisposition and exposure to environmental factors, leading to deficient insulin production and secretion and the need for exogenous insulin [1,2]. However, the various pathophysiological factors are present to varying degrees, resulting in multiple presentations of the disease with different degrees of progression of pancreatic beta-cell damage and insulin deficiency [3,4].

In pancreatic beta cells, the precursor proinsulin is cleaved into C-peptide and insulin, secreted in equimolar proportions. The measurement of C-peptide is the best way to assess endogenous insulin distribution, as it does not undergo significant clearance during the first pass through the liver [5]. However, in patients with T1D, due to the progressive loss of beta cells, their production tends to be progressively reduced and may reach undetectable levels [6]. Since C-peptide has a longer half-life, its dosage has become a more stable and safer means of assessing pancreatic reserve [7], and it is used for both diagnosis [8] and prognosis [9] of the disease in patients. A recent study has evaluated C-peptide levels as a key component in identifying different subtypes or “endotypes” of T1D [3]. This may be useful for targeting treatment and preventing disease progression [10].

Emerging strategies to intervene in the progression of T1D focus on increasing the survival rate of residual β cells, which can be assessed by C-peptide secretion [11]. Therefore, it is important to investigate the characteristics associated with the reduction in pancreatic dysfunction in patients with T1D exhibiting substantial genomic diversity, aiming to contribute to the understanding of the different phenotypes of this complex pathology. A previous study has demonstrated that genetic ancestry affects the secretion of C-peptide, particularly its increase, in patients with type 2 diabetes or glucose intolerance [12]. To the best of our knowledge, no assessment has been conducted to determine the relationship between the percentage of genomic ancestry and residual C-peptide secretion in patients with T1D.

The DCCT (Diabetes Control and Complications Trial) study demonstrated a correlation between C-peptide levels and glycemic control. Patients with higher levels of C-peptide (an indicator of endogenous insulin production) exhibited superior metabolic control and a diminished risk of chronic complications associated with diabetes [13]. The maintenance of detectable levels of C-peptide in patients with T1D is associated with specific variables, including the age at diagnosis, the duration of the illness, the type of assay utilized, and the timing of the sample collection (fasting or after stimulation, such as a mixed meal, for example) [14].

In this context, the evidence indicates that individuals diagnosed at a younger age tend to exhibit lower levels of C-peptide compared to those diagnosed at an older age. This phenomenon is also observed in patients with a shorter duration of illness than in individuals diagnosed longer ago [7]. Nevertheless, there remains a paucity of consensus in the literature regarding the utilization of C-peptide levels for the diagnosis and prediction of outcomes in patients with T1D. As proposed by Jones and Hattersley [5] in their review, a serum level of less than 0.75 ng/dL has been suggested as a potential indicator of T1D. Moreover, the 50-year Joslin Medalist study classified patients with levels below 0.09 ng/dL as having undetectable C-peptide [9].

Therefore, the aim of the present study is to investigate the relationship between C-peptide levels, clinical features, serum data, and genomic ancestry percentage in a highly admixed T1D sample from patients attending a specialized public diabetes outpatient clinic in a northeastern city in Brazil.

## 2. Results

A total of 95 patients (53 females and 42 males) with a mean age of 28.8 ± 10.7 years were included in this study. The general characterization data in Table 1 show that patients who were 10 years of age or younger when first diagnosed with T1D were most common in the sample (40%), and 42.1% were diagnosed with T1D when they were between 5 and 10 years old. It was observed that 22.6% and 4.3% of the sample were overweight and obese, respectively, according to the BMI measurements, with a predominance of eutrophic patients (60.2%). In addition, 29.9% were diagnosed with retinopathy, and 17.6% had albuminuria detected on examination.

Table 2 shows the distribution of the serum laboratory data. For C-peptide, 70.5% had a concentration of <0.10 ng/mL, 11.6% between 0.10 and 0.24 ng/mL, and 17.9% between 0.25 and 0.75 ng/mL. The mean fasting blood glucose was 194 ± 113, 28.4% of patients had an Hb1ac of >10%, and the mean total insulin dose and insulin dose/kg were 49.2 ± 19.1 and 0.82 ± 0.26, respectively. Regarding the lipid profile markers, 21.7% of patients had altered total cholesterol, 58.9% had altered HDL, 12.6% had borderline/high LDL, and 11.6% had altered triglyceride levels. A CRP higher than 0.3 mg/dL was observed in 28.5% of patients. The distribution of the other markers is shown in the table.

Table 3 shows the analysis of the relationship between general factors and the level of C-peptide in the sample. Low C-peptide levels were observed more frequently in patients aged 20–29 years (85.7%) compared to the other age groups and were statistically more frequent in the group of patients diagnosed before 10 years of age (*p* = 0.016). In addition, the mean age at first diagnosis of T1D was statistically lower in the <0.10 ng/dL peptide category (13.0 ± 9.1 vs. 19.7 ± 10.8; *p* = 0.002). There were no statistically significant differences in microvascular complications (retinopathy and albuminuria), abdominal circumference measurements, or blood pressure levels.

In the analysis of serum marker categories, no significant associations were found with the C-peptide levels (Table 4 and Table 5).

Table 6 shows that the mean age at diagnosis was lower in the group of patients with C-peptide levels of <0.10 than in the group with C-peptide levels between 0.25 and 0.75 ng/mL (13.0 ± 9.1 years vs. 18.9 ± 12.5 years; *p* = 0.028).

**Figure 1:**
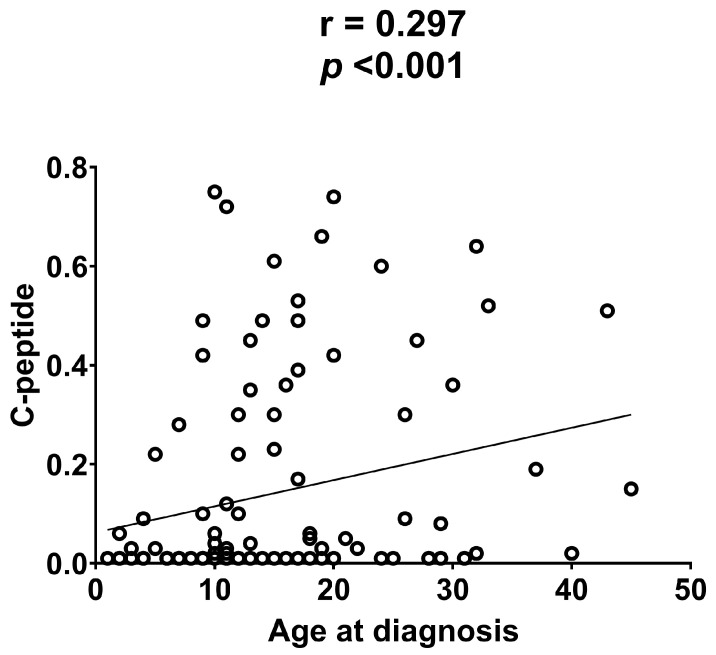
Scatterplot of the correlation analysis between the level of C-peptide and the age at diagnosis of type 1 diabetes. The values for an individual data point are indicated by the position of each dot on the horizontal and vertical axes. The line represents the linear correlation trend between the variables.

In addition, a weak direct correlation was observed between the age at diagnosis and C-peptide (r = 0.297; *p* < 0.001) (Figure 1).

Table 7 shows the distribution of genomic ancestry in the study sample. Overall, European ancestry had the highest mean percentage (47.7 ± 16.4), followed by African (27.9 ± 13.3), and Native American (24.3 ± 11.3). There were no significant differences in the percentages of the three ancestries according to the C-peptide levels.

## 3. Discussion

The present study has demonstrated that approximately 30% of patients exhibited residual insulin secretion. Additionally, a direct proportional relationship was identified between the serum C-peptide level and the age at diagnosis. However, no statistically significant differences were observed with regard to disease control or microvascular complications. Additionally, in our highly admixed population, ancestry percentage did not affect the persistence of residual C-peptide function. This suggests that further research based on genomic ancestry is warranted in other admixed populations with T1D to corroborate our results.

The majority of T1D diagnoses occurred before the age of 19 years (70.5%), a finding that has been corroborated by previous research. The SEARCH for Diabetes in Youth study group, which assessed the prevalence of types 1 and 2 diabetes in young people in the USA between 2001 and 2017, confirmed two peaks in the incidence of T1D diagnosis, including in middle childhood (predominantly after the age of 4 years) and between 10 and 14 years old. Additionally, the study group observed an upward trend in the estimated prevalence of T1D in patients under 19 years old [15].

It was observed that 29.5% of the patients exhibited a C-peptide level of ≥0.10 ng/mL, indicative of residual insulin release. This result is consistent with the findings of a study conducted by Davis and colleagues, in which 29% of the participants exhibited detectable C-peptide levels during fasting periods [16]. In a study that investigated C-peptide levels in Chinese patients diagnosed at least ten years prior, 38.5% of those assessed exhibited residual levels while fasting [17].

A previous study has reported that the pattern of miscegenation in the Maranhão population differs from the average found in the rest of Brazil. The Maranhão population has a lower percentage of European ancestry (46.5% vs. 68.1%) and a higher rate of African and Native American ancestry (approximately 25% each, in contrast to 19.6% African and 11.6% Native American in the Brazilian sample overall) [18]. Our assessment of the genomic ancestry of T1D in our sample revealed a comparable pattern, with 47.7% of the ancestry attributed to European, 24.3% to Native American, and 27.9% to African origins. The influence of genomic ancestry percentages on the residual function of C-peptide was not found to be statistically significant. However, Sjaarda et al. reported that there is an effect, particularly of African ancestry, on increased C-peptide secretion with an increased risk of type 2 diabetes and insulin resistance [12]. In patients with T1D, where there is an absence of insulin, no difference in C-peptide levels was observed based on genomic ancestry. This contrasts with the situation in DM2 patients, who are characterized by hyperinsulinemia and in whom differences in C-peptide levels based on genomic ancestry have been reported.

In evaluating the age at diagnosis and C-peptide levels, a reference value of ≥0.1 ng/mL was employed to define residual secretion, in accordance with the methodology utilized in the 50-year Joslin Medalist study [9]. The initial division of patients into two groups, based on C-peptide levels of <0.1 and ≥0.1 ng/mL, revealed a statistically significant difference, with values of <0.1 ng/mL being associated with a lower mean age at diagnosis. A more detailed analysis of secretion capacity revealed a directly proportional relationship between the age at diagnosis and the C-peptide level when the latter was divided into three subgroups (C-peptide levels of <0.1, between 0.1 and 0.24, and between 0.25 and 0.75 ng/mL). In a European multicenter longitudinal study, Barker et al. [19] demonstrated a correlation between lower C-peptide levels and a younger age at diagnosis. Similar results were observed in American research conducted by Wang et al. [20] and in works published by Gabbay et al. [21] and by the DCCT/EDIC group [7]. It is proposed that this directly proportional relationship between the age at diagnosis and the C-peptide level can be explained by a more intense autoimmune process in individuals diagnosed at a younger age. As observed by Leete et al. [22], patients diagnosed before seven years of age exhibited a more pronounced inflammatory process compared to those diagnosed at over 12 years of age. This resulted in the proposal of dividing the patients into two distinct endotypes, designated as 1 and 2, based on the age of onset and the intensity of pancreatic injury. In a recent study, the age at diagnosis and the C-peptide level were again used to propose grouping into other endotypes, in addition to the presence of risk HLA haplotypes, autoantibodies, and body mass index [23].

The present study found no statistically significant association between C-peptide level and disease duration, which differs from the results of recently published national research [21] and corroborates the findings of the Joslin Medalist study [8]. This discrepancy may be attributed to the fact that the average time from diagnosis exceeded 12 years in our research and in the Joslin Medalist study. It is presumed that this result is due to the prolonged exposure to autoimmune attack against β cells.

The DCCT (Diabetes Control and Complications Trial) study demonstrated a correlation between elevated C-peptide levels and enhanced metabolic control. However, the 25-year follow-up of the Diabetes Complications (EDC) study [9] and the research developed by Serfaty et al. [24] demonstrated no statistically significant difference in HbA1C levels and in the persistence of the residual function of pancreatic beta cells. Furthermore, our study did not reveal any statistically significant differences in glycemic control.

In the study conducted by Kuhtreiber et al. [25], patients with C-peptide levels below 0.03 ng/mL were 3.1 times more likely to develop a diabetic complication than those with levels above 0.03 ng/mL (odds ratio 3.1, confidence interval 1.1–8.6). However, this analysis revealed an association between C-peptide levels and glycemic control. A prospective study by Williams et al. [9] and research by Keenan et al. [8] revealed no statistically significant correlation between C-peptide and microvascular complications. The present study did not find a statistically significant association between C-peptide levels and microvascular complications, including retinopathy and nephropathy. Additionally, no association was identified between C-peptide levels and glycemic control. These findings diverge from those of other studies, which have indicated that elevated C-peptide levels, a marker of endogenous insulin secretion, are associated with enhanced glycemic control and a reduced risk of complications. It is plausible that the absence of an association between C-peptide and glycemic control may have contributed to these disparate outcomes. In contrast with our findings, a recent national survey [21] reported a correlation between C-peptide and albuminuria. However, in contrast with the aforementioned study, this association was independent of glycemic control. The authors postulated a potential link with lower glycemic variability, as observed in a Chinese study [26].

A limitation of the present study is its cross-sectional design, which requires careful consideration of the potential causal direction between the observed associations. In addition, the small sample size may have contributed to the failure to detect significant differences due to power limitations. On the other hand, the present study seems to be the first to investigate the relationship between C-peptide levels and genomic ancestry markers in a highly admixed T1D population from Brazil. Therefore, future studies with longitudinal follow-up design, with larger and other admixed samples, should be performed to support the present findings.

## 4. Materials and Methods

### 4.1. Study Design

This cross-sectional study was conducted at the Endocrinology Service of the University Hospital of the Federal University of Maranhão (HU-UFMA). Patients who met the inclusion criteria and agreed to participate were enrolled after reading and signing the free and informed consent form. This study was approved by the ethics committee of the HUUFMA under opinion number 59795116.9.0000.5086. The study population consisted of patients with T1D from the diabetes outpatient clinics of HU-UFMA. Patients with T1D diagnosed at least five years previously were included, and those with a history of acute infectious diseases or diabetic ketoacidosis, pregnancy, and lactation in the three months prior to the evaluation were excluded.

### 4.2. General Health Data

A clinical demographic survey was conducted with patients using a standardized questionnaire. Data were collected on sex, age (in years), age at diagnosis (in years), and duration of T1D (in years). Clinical assessments included the measurement of weight (in kg), height (in cm), and body mass index (BMI).

### 4.3. Serum Data Collection

Glycated hemoglobin (A1c) levels were quantified using high-performance liquid chromatography (HPLC), with a reference range of 4.0–6.0%. Albuminuria was quantified by immunoturbidimetry on isolated urine samples. C-peptide levels were quantified by the electrochemiluminescence method (Elecsys C-Peptide-Cobas) with a reference value of 1.1–4.4 ng/mL (range: 0.010–40.0 ng/mL) using fasting samples. Patients were classified as having T1D if their C-peptide levels were less than 0.75 ng/mL, and as not having T1D if their levels were less than 0.1 ng/mL.

### 4.4. Retinopathy Evaluation

Diabetic retinopathy was assessed by ophthalmoscopy using indirect ophthalmoscopy (Eyetech^®^, São Carlos, Brazil) under the effect of topical mydriatic medication and classified as normal non-proliferative retinopathy, proliferative retinopathy, and maculopathy, with the most affected eye being used for diagnosis.

### 4.5. Kidney Function Evaluation

Diabetic kidney disease was assessed using estimated glomerular filtration rate (CKD-EPI equations) and urinary albumin concentration. Albuminuria was defined as positive if the urinary albumin concentration was greater than or equal to 30 mg/L.

### 4.6. Genetic Ancestry Percentage

DNA was extracted from peripheral blood using a commercial SP QIA Symphony kit (Qiagen, Germantown, MD, USA) following the manufacturer’s standard instructions. Genomic ancestry was evaluated using a genetic panel comprising 46 informative autosomal insertion/deletion ancestry markers (AIM-indels). The aforementioned markers were amplified in a single multiplex PCR, following the protocol described by Pereira et al. [27]. The polymorphisms present in the generated fragments were identified through capillary electrophoresis using an ABI 3500 automated sequencer (Life Technologies Corporation, Carlsbad, CA, USA). Genotyping was conducted using GeneMapper Analysis v.4.1 software (Applied Biosystems, Foster City, CA, USA). Ancestry prediction was conducted using Structure v.2.3.3 software. The percentages of African, European, and Native American ancestry were estimated to add up to 100% for each subject.

### 4.7. Statistical Analysis

Data analysis was performed using the R statistical package, version 4 (R Foundation for Statistical Computing, Vienna, Austria) and GraphPad Prism, version 10.0 (GraphPad Software Inc., San Diego, CA, USA). First, descriptive statistics were calculated using absolute, relative, mean, and standard deviation (±SD) frequency measures. The chi-squared test, or Fisher’s exact test, was used to compare the categorical data. The normality of the distribution of continuous variables was assessed using the Shapiro–Wilk test. As non-normal distributions were mostly detected, the non-parametric Mann–Whitney test was used to compare continuous data between the two C-peptide categories (<0.10 and ≥0.10). One-way ANOVA was used to compare the age at and time since T1D diagnosis in three level categories. Pearson’s coefficient was calculated to assess the correlation between the age at T1D diagnosis and C-peptide levels. For all analyses, the significance level was set at 5% (*p* < 0.05).

## 5. Conclusions

The study findings indicate a direct correlation between the age at diagnosis and C-peptide levels in T1D patients. Residual pancreatic beta-cell function was not associated with better disease control or the absence of microvascular complications in the study sample. Additionally, our results suggest that ancestry does not appear to be related to the mechanisms influencing the rate of beta-cell destruction and insulin secretion in a highly admixed T1D population in Brazil. It is recommended that future studies consider including additional variables, such as HLA and pancreatic autoimmunity, as potential influencers of residual β-cell function.

## Figures and Tables

**Table 1:** Demographic and clinical characteristics distribution of the sample.

Variables	Mean	±sd	*n*	(%)
Sex				
Female			53	(55.8)
Male			42	(44.2)
Age group	28.8	±10.7		
Up to 19 years old			21	(22.1)
20 to 29 years old			35	(36.9)
30 to 39 years old			21	(22.1)
40 years or older			18	(18.9)
Age at T1D diagnosis	15.1	±10.1		
≤10 years old			38	(40.0)
11–18 years old			29	(30.5)
≥19 years old			28	(29.5)
Time since T1D diagnosis	13.9	±7.2		
5–10 years old			40	(42.1)
11–15 years old			20	(21.1)
16–20 years old			21	(22.1)
>20 years			14	(14.7)
Body mass index (kg/m^2^)	22.9	±3.6		
<18.5 (Underweight)			12	(12.9)
18.5 to24.9 (Normal)			56	(60.2)
25 to 29.9 (Overweight)			21	(22.6)
>30 (Obese)			4	(4.3)
Abdominal circumference (cm)	80.3	±11.4		
Systolic blood pressure (mmHg)	118.0	±15.4		
Diastolic blood pressure (mmHg)	74.3	±10.1		
Retinopathy				
No			61	(70.1)
Yes			29	(29.9)
Albuminuria				
<30			75	(82.4)
≥30			16	(17.6)

±sd = standard deviation. T1D = type 1 diabetes.

**Table 2:** Distribution of laboratory variables in the study sample.

Variables	Mean	±sd	*n*	(%)
C-peptide (ng/mL)				
<0.10			67	(70.5)
0.10 to 0.24			11	(11.6)
0.25 to 0.75			17	(17.9)
Fasting blood glucose (mg/dL)	194	±113		
Hb1ac	9.1	±2.2		
<7%			17	(17.9)
7 to 10%			51	(53.7)
>10%			27	(28.4)
Insulin total dose (UI)	49.2	±19.1		
Insulin dose per weight (UI/kg)	0.82	±0.26		
Total cholesterol (mg/dL)	169.0	±40.5		
Normal			78	(82.1)
Altered (>200)			17	(17.9)
HDL (mg/dL)	54.2	±14.4		
Altered (<50)			39	(41.1)
Normal			56	(58.9)
LDL (mg/dL)	93.8	±28.4		
Optimal (<130)			83	(87.4)
Borderline/High (≥130)			12	(12.6)
Triglycerides (mg/dL)	98.9	±57.4		
Normal			84	(88.4)
Altered (>150)			11	(11.6)
C-reactive protein (mg/dL)	0.31	±0.45		
<0.01			35	(41.7)
0.01 to 0.03			25	(29.8)
>0.03			24	(28.5)
Hemoglobin (g/dL)	13.6	±1.8		
Leucocytes (mil/mm^3^)	6.1	±1.6		
Urea (mg/dL)	30.9	±15.5		
Creatinine (mg/dL)	1.35	±4.70		
AST (mg/dL)	20.9	±7.7		
ALT (mg/dL)	20.4	±15.4		

±sd = standard deviation. Hb1ac = glycated hemoglobin. HDL = high-density lipoproteins. LDL = low-density lipoproteins. AST = glutamic oxaloacetic transaminase. ALT = glutamic pyruvic transaminase.

**Table 3:** Comparative analysis of demographic and clinical data between C-peptide level categories.

Variables	Level of C-Peptide (ng/mL)				*p*
	<0.10 ng/mL		≥0.10 ng/mL		
	*n*	(%)	*n*	(%)	
Sex					0.463
Female	39	(73.6)	14	(26.4)	
Male	28	(66.7)	14	(33.3)	
Age group					0.065
Up to 19 years old	14	(66.7)	7	(33.3)	
20 to 29 years old	30	(85.7)	5	(14.3)	
30 to 39 years old	13	(61.9)	8	(38.1)	
40 years or older	10	(55.6)	8	(44.4)	
Age at T1D diagnosis					0.016 *
≤10 years old	33	(86.8)	5	(13.2)	
11–18 years old	18	(62.1)	11	(37.9)	
≥19 years old	16	(57.1)	12	(42.9)	
Time since T1D diagnosis					0.856
5–10 years old	27	(67.5)	13	(32.5)	
11–15 years old	15	(75.0)	5	(25.0)	
16–20 years old	14	(66.7)	7	(33.3)	
>20 years	11	(78.6)	3	(21.4)	
Retinopathy					0.446
No	42	(68.9)	19	(31.1)	
Yes	20	(76.9)	6	(23.1)	
Albuminuria					0.794
<30	54	(72.0)	21	(28.0)	
≥30	11	(68.8)	5	(31.3)	
	**mean**	**±sd**	**mean**	**±sd**	
Age at diagnosis	13.0	±9.1	19.7	±10.8	0.002 *
AC (cm)	80.2	±12.0	80.5	±10.2	0.842
SBP (mmHg)	117.5	±16.3	118.2	±13.3	0.833
DBP (mmHg)	74.9	±10.1	72.7	±10.2	0.328

±sd = standard deviation. T1D = type 1 diabetes. AC = abdominal circumference. SBP = systolic blood pressure. DBP = diastolic blood pressure. * Indicates statistically significant differences (*p* < 0.05).

**Table 4:** Comparative analysis of laboratory data between C-peptide level categories.

Variables	Level of C-Peptide (ng/mL)				*p*
	<0.10 ng/mL		≥0.10 ng/mL		
	Mean	±sd	Mean	±sd	
Fasting blood glucose (mg/dL)	190.3	±119.4	202.3	±97.7	0.369
Hb1ac (%)	9.1	±2.0	8.9	±2.7	0.398
Insulin total dose (UI)	51.2	±18.3	44.2	±20.1	0.077
Insulin dose/weight (UI/kg)	0.85	±0.25	0.77	±0.28	0.159
Total cholesterol (mg/dL)	169.9	±35.6	165.9	±50.9	0.361
HDL (mg/dL)	55.7	±13.9	50.8	±15.2	0.091
LDL (mg/dL)	94.9	±26.3	91.4	±33.4	0.400
Triglycerides (mg/dL)	97.9	±57.3	101.2	±58.5	0.590
C-reactive protein (mg/dL)	0.32	±0.46	0.26	±0.42	0.717
Hemoglobin (g/dL)	13.5	±1.8	13.7	±1.9	0.958
Leucocytes (mil/mm^3^)	6.1	±1.6	6.1	±1.7	0.698
Urea (mg/dL)	30.1	±15.7	32.7	±15.1	0.534
Creatinine (mg/dL)	1.60	±5.60	0.76	±0.17	0.290
AST (mg/dL)	20.7	±8.3	21.3	±5.7	0.243
ALT (mg/dL)	18.3	±9.1	25.8	±25.2	0.120

±sd = standard deviation. Hb1ac = glycated hemoglobin. HDL = high-density lipoproteins. LDL = low-density lipoproteins. AST = glutamic oxaloacetic transaminase. ALT = glutamic pyruvic transaminase.

**Table 5:** Distribution of laboratory data categories according to C-peptide level.

Variables	Level of C-Peptide (ng/mL)				*p*
	<0.10 ng/mL		≥0.10 ng/mL		
	*n*	(%)	*n*	(%)	
Hb1ac					0.191
<7%	9	(52.9)	8	(47.1)	
7 to 10%	37	(72.5)	14	(27.5)	
>10%	21	(77.8)	6	(22.2)	
C-reactive protein					0.996
<0.01	25	(71.4)	10	(28.6)	
0.01 to 0.03	18	(72.0)	7	(28.0)	
>0.03	17	(70.8)	7	(29.2)	
Total cholesterol (mg/dL)					0.995
Normal	55	(70.5)	23	(29.5)	
Altered (>200)	12	(70.6)	5	(29.4)	
HDL (mg/dL)					0.109
Altered (<50)	24	(61.5)	15	(38.5)	
Normal	43	(76.8)	13	(23.2)	
LDL (mg/dL)					0.744
Optimal (<130)	59	(71.1)	24	(28.9)	
Bordeline/High (≥130)	8	(66.7)	4	(33.3)	
Triglycerides (mg/dL)					1.000
Normal	59	(70.2)	25	(29.8)	
Altered (>150)	8	(72.7)	3	(27.3)	

Hb1ac = glycated hemoglobin. HDL = high-density lipoproteins. LDL = low-density lipoproteins.

**Table 6:** Comparative analysis of mean age at diagnosis and disease duration between C-peptide categories.

Variables	Level of C-Peptide (ng/mL)						*p*
	<0.10		0.10 to 0.24		0.25 to 0.75		
	Mean	±sd	Mean	±sd	Mean	±sd	
Age at T1D diagnosis	13.0	±9.1 ^a^	18.9	±12.5 ^ab^	20.2	±9.8 ^b^	0.028 *
Time since T1D diagnosis	14.0	±7.3	14.8	±6.7	12.5	±7.6	0.689

* Statistically significant differences (*p* < 0.05). Different superscript letters indicate significant differences between categories.

**Table 7:** Comparative analysis of the percentage of African, European, and Native American genetic ancestry according to C-peptide levels.

Ancestry Percentage	Overall		Level of C-Peptide (ng/mL)				*p*
			<0.10 ng/mL		≥0.10 ng/mL		
	Mean	±sd	Mean Rank	Sum of Ranks	Mean Rank	Sum of Ranks	
African	27.9	±13.3	44.3	2838	48.3	1256	0.513
European	47.7	±16.4	46.4	2974	43.1	1120	0.578
Native American	24.3	±11.3	45.9	2938	44.5	1157	0.817

±sd = standard deviation.

## Data Availability

The original contributions presented in this study are included in the article, and further inquiries can be directed to the corresponding authors.
